# Assessing the Link between Diabetic Metabolic Dysregulation and Breast Cancer Progression

**DOI:** 10.3390/ijms241411816

**Published:** 2023-07-23

**Authors:** Samrein B. M. Ahmed, Nada Radwan, Sara Amer, Narjes Saheb Sharif-Askari, Amena Mahdami, Kamel A. Samara, Rabih Halwani, Herbert F. Jelinek

**Affiliations:** 1 Research Institute of Medical and Health Sciences, University of Sharjah, Sharjah 27272, United Arab Emiratesmd_saleema@outlook.com (S.A.); rhalwani@sharjah.ac.ae (R.H.); 2 College of Medicine, University of Sharjah, Sharjah 27272, United Arab Emirates; 3 College of Health, Wellbeing and Life Sciences, Department of Biosciences and Chemistry, Sheffield Hallam University, Sheffield S1 1WB, UK; 4 Department of Biomedical Engineering and Health Engineering Innovation Center, Khalifa University, Abu Dhabi 127788, United Arab Emirates

**Keywords:** breast cancer, diabetes, hyperglycemia, hyperinsulinemia, gene dysregulation

## Abstract

Diabetes mellitus is a burdensome disease that affects various cellular functions through altered glucose metabolism. Several reports have linked diabetes to cancer development; however, the exact molecular mechanism of how diabetes-related traits contribute to cancer progression is not fully understood. The current study aimed to explore the molecular mechanism underlying the potential effect of hyperglycemia combined with hyperinsulinemia on the progression of breast cancer cells. To this end, gene dysregulation induced by the exposure of MCF7 breast cancer cells to hyperglycemia (HG), or a combination of hyperglycemia and hyperinsulinemia (HGI), was analyzed using a microarray gene expression assay. Hyperglycemia combined with hyperinsulinemia induced differential expression of 45 genes (greater than or equal to two-fold), which were not shared by other treatments. On the other hand, in silico analysis performed using a publicly available dataset (GEO: GSE150586) revealed differential upregulation of 15 genes in the breast tumor tissues of diabetic patients with breast cancer when compared with breast cancer patients with no diabetes. *SLC26A11, ALDH1A3*, *MED20*, *PABPC4* and *SCP2* were among the top upregulated genes in both microarray data and the in silico analysis. In conclusion, hyperglycemia combined with hyperinsulinemia caused a likely unique signature that contributes to acquiring more carcinogenic traits. Indeed, these findings might potentially add emphasis on how monitoring diabetes-related metabolic alteration as an adjunct to diabetes therapy is important in improving breast cancer outcomes. However, further detailed studies are required to decipher the role of the highlighted genes, in this study, in the pathogenesis of breast cancer in patients with a different glycemic index.

## 1. Introduction

Aberrant glucose metabolism has been linked to poor breast cancer outcomes [1,2]. It is well established that diabetic/hyperglycemic metastatic cancer patients have a decreased overall survival compared to a nondiabetic/normoglycemic control group [3]. Previous reports have proposed that glycemic control medications can be used as an adjunct to chemotherapy, claiming that the glycemic index is related to metabolic alterations in cancer patients [4,5,6]. However, overall, diabetic women with breast cancer demonstrated poorer outcomes than non-diabetic women with breast cancer. The cause of this disparity has not yet been identified and needs to be determined.

**Figure 1:**
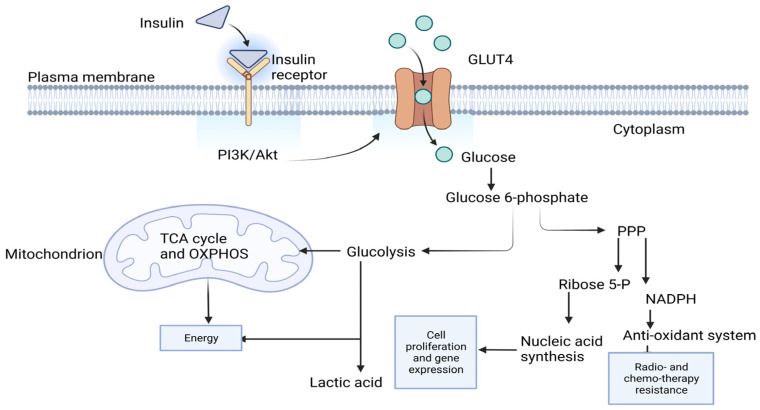
Glucose metabolism. In response to insulin, Phosphoinositide 3-kinase (PI3K)/Akt pathway is stimulated; this leads to glucose transporter 4 (GLUT4) translocating to the membrane. GLUT4 allows the influx of glucose. Glucose is first phosphorylated at carbon 6 to give glucose 6-phosphate, which is going to act as a precursor to glycolysis and pentose phosphate pathway (PPP). Glycolysis gives 2 pyruvate molecules that are going to be decarboxylated to result in 2 acetyl-CoA molecules. Acetyl-CoA is the main driver for tricarboxylic cycle (TCA) that produces NADH and FADH_2_. NADH and FADH_2_ join oxidative phosphorylation (OXPHOS) to produce paramount energy. Glycolysis produces less energy production and lactic acid. PPP is the pathway that produces ribose 5-phospahate, which is key in nucleotide biosynthesis. Nucleotides are the building blocks for nucleic acids, which are the vital element in cell proliferation and gene expression. PPP also produces NADPH, which is important in glutathione antioxidant system. Glutathione system upregulation is one of the resistant mechanisms adopted by cancer cells to resist radio- and chemotherapy. The diagram is adapted from [14] and created with BioRender.com.

Glucose, for cancer cells, is used not only to satisfy energy needs but also to provide precursor molecules that are vital for other biological needs such as cell proliferation. Glucose uptake by cells is determined by a group of glucose transporters that vary in their expression among different body cells. Breast cancer cell lines such as MCF7 and MDA-MB-231 depend on glucose transporter 4 (GLUT4) in their basal glucose uptake [7]. GLUT4 is the key factor in glucose transport and homeostasis in response to insulin [8] (Figure 1). Upon the uptake of glucose, glucose is phosphorylated to give glucose 6-phosphate/P, which is the forerunner for glycolysis and pentose phosphate pathway (PPP) [9] (Figure 1). Glucose 6-P is metabolized to two pyruvate molecules via the glycolytic pathway [10]. Pyruvate is then decarboxylated to acetyl-CoA, which is the driver molecule for the tricarboxylic cycle (TCA) [10]. NADH and FADH_2_ generated by the TCA cycle join oxidative phosphorylation to help in ATP production. Glucose 6-P is also utilized by PPP to produce ribose 5-P and NADPH [11]. The former is crucial for nucleic acids synthesis, which is important to fulfill the high demand of cancer cells for proliferation and gene expression. The latter is valuable in the glutathione antioxidant system that is adopted by cancer cells to resist radio- and chemotherapy [12] (Figure 1). With the presence of oxygen, some cancer cells rewire their metabolism and rely on glycolysis as a main energy provider through the ‘Warburg effect’; in this way, they upregulate their glucose uptake system to ensure the availability of glucose to support their oncogenic behavior [13].

In vitro studies have demonstrated that treating breast cancer and pancreatic cancer cells with high glucose results in molecular changes such as phosphorylation of the epidermal growth factor receptor (EGFR), which promotes cancer cell proliferation [15,16]. Further to that, a study by Lopez et al. showed that hyperglycemia activated the AKT/mTOR pathway and impacted cell proliferation [17].

A study by Flores et al. also revealed that high concentrations of glucose promoted cell proliferation in MDA-MB-231 breast cancer cells when compared with cells treated with a low concentration of glucose [18]. An additional report showed that under hyperglycemic conditions, neutrophil mobilization was inhibited; therefore, tumor cells were able to evade the immune system, resulting in tumor growth enhancement and metastatic abilities [19]. Moreover, long-term hyperglycemia led to an enhancement in the expression of some pro-inflammatory factors such as IL6, COX2 and TNFα, which were proven to be linked to tumorigenic behaviour such as triggering an anti-apoptotic signal and promoting epithelial mesenchymal transition [20]. An interesting study by Li et al. revealed the role of hyperglycemia in activating ERK via H_2_O_2_ production associated hyperglycemia [21]. Hyperglycemia-induced ERK was linked to increased tumour migration and invasion. Further, high glucose levels during chemotherapy contributed to the development of chemoresistance by tumour cells [22].

In most of the previous studies, hyperglycemia was considered as the main metabolic factor affecting tumor cell proliferation and migration, neglecting the fact that diabetes is a multi-organ disease associated with an array of metabolic changes involving hyperglycemia and hyperinsulinemia. Hyperinsulinemia has been shown to predispose to various diseases such as cardiovascular disease, hypertension, stroke and cancer [23]. It is worth mentioning, in comparison to the few in vitro studies in which hyperglycemia was combined with hyperinsulinemia [24,25], that the current study provides a novel global-gene approach, using a DNA microarray, to identify the dysregulated genes and metabolic alterations.

Since diabetes and breast cancer are global health concerns among females [26,27,28], we believe that the study improves our understanding of how metabolic changes associated with diabetes affect tumor function and tumor progression. Moreover, the outcome of this study suggests a greater emphasis on personalized medicine.

## 2. Results

### 2.1. Differentially Expressed Genes in MCF7 with Different Treatment

The differentially expressed genes under normoglycemia (NG), normoglycemia with hyperinsulinemia (NGI), hyperglycemia (HG) or hyperglycemia with hyperinsulinemia (HGI) are available in the Supplementary Materials. Comparative analysis was conducted between NG vs. HG, NG vs. NGI and NG vs. HGI. The NG vs. HGI comparison revealed 245 upregulated genes (*p* < 0.05) and 491 downregulated genes (*p* < 0.05). Only the genes with a *p* value <0.05 and two-fold increase were considered in this report (Table 1). After applying the inclusion criteria, HG treatment resulted in upregulation of 16 genes, and examples of these genes are: *TGFA*, *CDCA5*, *MUC3A* and *C3AR1* (Supplementary Table S2). HGI caused upregulation of 57 genes including *TGFA*, *CDCA5* and *C3AR1*, which were also noted with HG treatment only (Table 1). Forty-five genes out of the fifty-seven genes were found to be exclusively upregulated with the combination of hyperglycemia and hyperinsulinemia. Example of these genes are: *FGFR3*, *PAK1*, *LEPR*, *SCP2*, *E2F7*, *AGER*, *ALDH1A3* and *DOCK7*. On the other hand, 25 nM insulin treatment per se had also positively influenced gene expression such as *TGFA*, *CDCA5*, *PCM1*, *DDIT3*, *ZC3H12D*, *MUC3A*, *AKR1B1* and *LIMD1* (Supplementary Table S3). The shared overexpressed genes between different treatments are listed in Supplementary Table S4.

**Figure 2:**
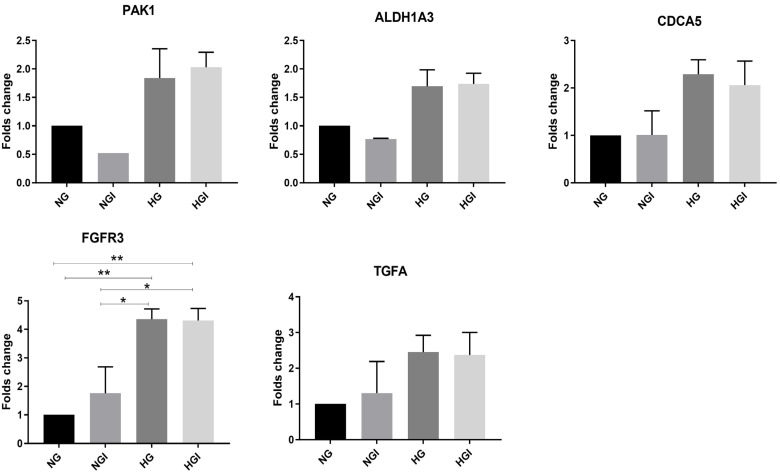
Validation of the microarray data by RT-PCR. MCF7 cells were either grown in 5.6 mM glucose (NG), 5.6 mM glucose and 20 pmol/L insulin (NGI), 30 mM glucose (HG) or 30 mM glucose and 20 pmol/L insulin (HGI) for 72 h. The cells were then harvested, and the RNA was extracted. After cDNA production, the RT-PCR was performed. For each sample, 18 S was used as an internal control. The fold change in the indicated genes in HG and HG1 is shown in comparison to NG and NGI, respectively. * < 0.05; ** < 0.01; ns: not significant. Each of the bar charts demonstrated a pool of 2–3 independent biological repeats. Results are presented as mean (±SEM) fold change.

To validate the microarray findings, five genes, which are related to cancer traits, were tested by qRTPCR; these genes are *TGFA*, *CDC5A*, *PAK1*, *FGFR3* and *ALDH1A3*. All these genes demonstrated upregulation with hyperglycemia and hyperglycemia with hyperinsulinemia. Consistent with the microarray data, *CDCA5* was overexpressed with the HG and HGI treatments, while no change was noted with NGI (Figure 2). In the microarray data, *TGFA* exhibited elevation in both NGI and HG but surprisingly not with HGI. *TGFA* mRNA levels, obtained from qRTPCR, demonstrated slight increments; however, they were not significant, with NGI and a greater increase with HG and HGI, which partially validates the data obtained from the microarray results. A significant increase in the expression of *FGFR3* (*p* < 0.01) was noted with HGI treatment consistent with the microarray data (Figure 2). Overall, microarray data showed differential upregulation of different genes with different treatments, although some genes were shared by either the two or three treatment conditions. Employing RT-PCR has relatively validated the data obtained from the microarray data.

### 2.2. The Overlapping Differentially Expressed Genes from In Vitro Experiments and In Silico Data

**Figure 3:**
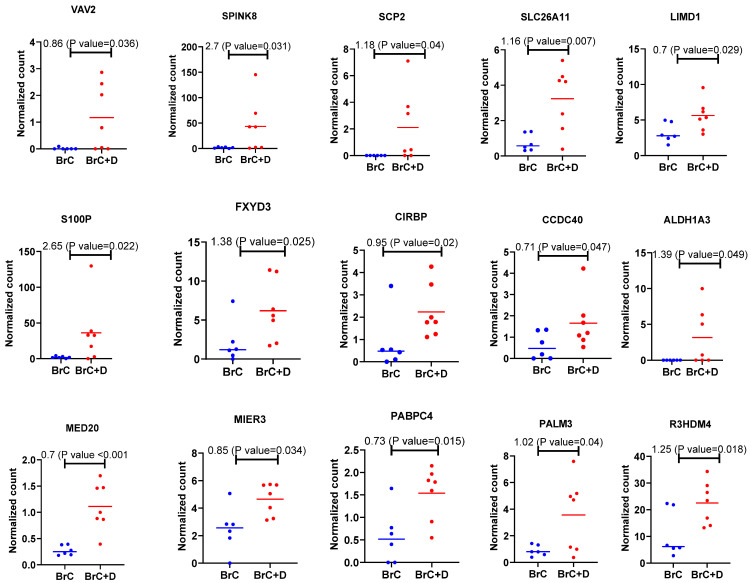
Differentially upregulated genes in breast cancer patients with diabetes vs. breast cancer patients with no diabetes. Control group (n = 6): breast tumor tissues obtained from patients with breast cancer with no diabetes; the test group (n = 7): breast cancer tissues obtained from breast cancer patients with diabetes. The data were processed by NovaSeq 6000. Data were analyzed by R software and Prism. *p* < 0.05 was considered significant. Results are presented as mean (±SEM) mRNA expression. BrC: breast cancer; BRC + D: breast cancer with diabetes.

It was of interest to find out whether diabetic patients with breast cancer show any overlapping results with the in vitro experiments. Differentially expressed genes obtained from a cohort of diabetic women with breast cancer (n = 7) and women with breast cancer (n = 6) with a normal glycemic index are plotted in Figure 3. Different genes were found to be upregulated in diabetic women with breast cancer. These genes are *VAV2*, *SPINK8*, *SCP2*, *SLC26A11*, *LIMD1*, *S100P*, *FXYD3*, *CIRBP*, *CCDC40*, *ALDH1A3*, *MED20*, *MTER3*, *PAPBPC4*, *PALM3* and *R3HDM4* (Figure 3).

**Figure 4:**
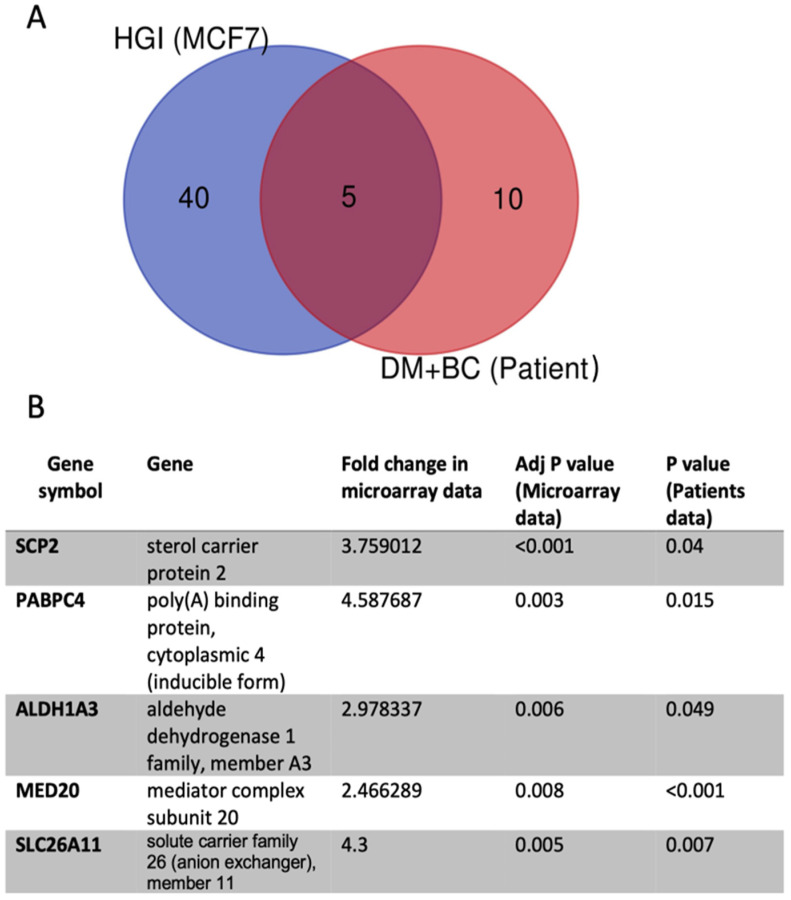
The Venn diagram shows the overlapping upregulated genes obtained from in vitro experimentation and in silico analysis of patients’ data. (**A**) Differentially upregulated genes in MCF7 treated with HGI (40 + 5 genes) (in the blue circle), differentially upregulated genes in breast cancer patients with diabetes (10 + 5 genes) (in the yellow circle) and the 5 overlapping genes in the intersected region. The Venn diagram was generated by Bioinformatics & Evolutionary Genomics tool [29]. (**B**) The coinciding genes between the HGI-related microarray data and publicly available data.

The overlapping upregulated genes, which are shared between the genes obtained from the microarray data (HGI-treated cells), and the genes obtained from the patient data are *SLC26A11*, *ALDH1A3*, *MED20*, *PABPC4* and *SCP2* (Figure 4).

## 3. Discussion

Breast cancer is one of the most common cancers among women worldwide [30]. The majority of breast cancer cases are sporadic, while a small percentage is due to hereditary factors [31]. The outcome of breast cancer is determined by different factors including: the stage and the grade of the disease, the expression of hormone receptors, the *HER2* receptor genes and age, among others [32]. The association of comorbidities, such as hypertension, pulmonary chronic obstructive disease, rheumatologic disease and diabetes mellitus with breast cancer has been shown previously [33,34]. Furthermore, the correlation of diabetes with a progressive form of breast cancer has also been described [35,36]. The main metabolic alterations that occur in diabetes are hyperglycemia and hyperinsulinemia. In this study, it was intended to investigate the effect of these metabolic alterations on the gene expression machinery employing the MCF7 breast cancer cell line. The exposure of these cells to hyperglycemia and hyperinsulinemia caused alterations in the gene transcription machinery. Publicly available data assisted in deciphering the differentially deregulated genes in diabetic breast cancer patients. The alignment between microarray data and the in silico analysis revealed *SLC26A11*, *ALDH1A3*, *MED20*, *PABPC4* and *SCP2* genes as upregulated on both datasets.

It is currently known that breast cancer has different molecular subtypes with different molecular signatures, which reflects the heterogeneity of breast cancer subtypes. Knowing the subtypes of breast cancer helps in determining which treatment the patient requires [37,38,39]. However, we believe determining certain chemotherapeutic regimen can be impacted by other factors, other than breast cancer molecular subtypes, such as high glucose levels and high insulin levels. The outcome of this current study hints towards glucose levels being an important factor not only in breast cancer progression but in other malignant cancers as well and therefore needs to be considered—when treating diabetic cancer patients.

Epidemiological studies revealed that hyperglycemia influences tumor progression in different types of cancer [35,36]. Hyperglycemia is commonly seen in diabetic patients as well as in diabetes-unrelated and related conditions such as metabolic syndrome, obesity, Cushing’s syndrome, hyperthyroidism, chronic stress, long-term prednisolone treatment and polycystic ovarian disease [40,41,42,43,44,45,46,47]. Furthermore, resistance to insulin results in an elevation in blood glucose as well as an increase in insulin levels. The combination of hyperglycemia and hyperinsulinemia is predominantly present in diabetes type 2 as well in metabolic syndrome [48]. Interestingly, hyperinsulinemia per se was reported to increase mortality in obese cancer patients [49]. It is worth noting that breast cancer is one of the most common occurring cancers among patients diagnosed with diabetes and metabolic syndrome [50,51,52]. Not only this, patients with these metabolic disorders, who develop cancer during the course of the chronic disease, exhibit a worse prognosis than those who have a normal glycemic index [1,35,49,50,51,52].

It is worth mentioning how tumor cells rewire their metabolism to fulfill their needs. Previously, it was shown that tumor cells have a high demand for glucose since they rely on anaerobic glycolysis (i.e., the Warburg effect) [53]; nevertheless, recently, it was revealed that tumor cells might also require oxidative phosphorylation to come on board to satisfy their energy demands in limited glucose conditions [54,55,56]. Moreover, shifting to oxidative phosphorylation has appeared to be a way to evade the killing effect of some anti-cancer agents [54,55]. Here, we are not limiting glucose conditions; we are instead offering tumor cells an excess of glucose to mimic the hyperglycemia that occurs in different metabolic diseases and looking at upregulated genes in the hyperglycemic state. Earlier reports focused on the effect of hyperglycemia, or hyperglycemia with hyperinsulinemia on certain signaling pathways [15,16,23,24]. Unlike previous studies, this study took a holistic approach to explore the effect of the combination of hyperglycemia and hyperinsulinemia in gene expression.

It is well studied that tumor cells feed on glucose and utilize it as a fuel to generate intermediates that are useful for cancer cell activities [57]. Therefore, the availability of glucose in the vicinity of the tumor cells, as well as the presence of insulin, leads to increased tumor progression. Previous studies showed that injecting mice with insulin triggered mammary tumor formation, while in rats, tumor-like pathologies were observed in the colon [58,59,60]. In an MKR transgenic mouse model, in which endogenous inulin receptors are inactivated in the skeletal muscles which results in hyperinsulinemia, IGFR, AKT and MAPK/ERK were found to be activated in the mammary tumor tissue in comparison to their control [58]. Hyperinsulinemia was also demonstrated to act as an inflammatory mediator. NF-κB and TNF α were found to be associated with insulin resistance [61,62].

Our microarray data revealed different upregulated genes with HGI treatment. Some of these genes contribute to cancer progression and development. According to our data, hyperglycemia alone caused upregulation of a number of genes such as *TGFA*, *CDCA5*, *MUC3A* and *C3AR1*. The *TGFA* levels were shown to be higher in breast cancer patients when compared with non-cancerous individuals [63], which indicates the role of TGFA in breast cancer carcinogenesis. TGFA was also linked to estrogen-receptor-expressing breast cancer, and its role in breast cancer progression was proposed [63]. Moreover, TGFA facilitates breast cancer metastasis to the bones [64]. On the other hand, treatment of vascular smooth muscle tissue with 30 mM of glucose resulted in a 1.7-fold increase in the expression of *TGFA* [65], which is in accordance with our data. Moreover, *CDCA5*, *MUC3A* and *C3AR1* gene overexpression has previously been linked to carcinogenesis; however, no association with the expression of these genes has been reported in relation to hyperglycemia and breast cancer [66,67,68,69,70]. The cell cycle division associated (CDCA) proteins family is known for its role in controlling the cell cycle [70]. The CDCA5 member is a regulator of sister chromatid separation during cell division. Its distinct expression was correlated with short relapse-free survival [70]. MUC3A encodes for a glycoprotein that is involved in mucin structure in the intestinal tract, while interestingly, its overexpression is linked to poor outcomes in renal cell carcinoma [66]. Furthermore, C3AR1 is a crucial regulator in complement signaling, and it is abundantly expressed in immune cells. The C3AR1 and C3a signaling axis is well documented in enhancing tumor metastasis via manipulating the tumor-associated fibroblasts [67]. The role of C3AR1 in tumor cells is not yet verified; although, its expression is associated with low survival rate in some cancers, as depicted by the Human Protein Atlas [71].

Among all the upregulated differentially expressed genes with HGI treatment, *SCP2*, *PABPC4*, *MED20*, *SLC26A11* and *ALDHA13* were found to coincide with data obtained from publicly available data. *SCP2* was reported to be upregulated in gliomas, and the levels of its expression were found to be correlated with glioma grades [72]. Although in this study we have not focused on *SCP2*, future work is planned to study the role of *SCP2* in breast cancer in relation to hyperglycemia and hyperinsulinemia. *MED20* belongs to a family of transcription factors that mediate transcription of different genes, including, importantly, the estrogen receptor gene among other intracellular receptors, which emphasize the role of this family in breast cancer pathogenesis [73,74]. Other MED genes such as *MED8* were found to associate with renal cell carcinoma, while *MED12* associates with lung cancer [73]. Furthermore, *MED1* was found to be linked to prostate cancer [75]. Intriguingly, multiomics analysis revealed *MED20* as one of the signatures related to tumor cells [76]. Hence, it could be that *MED20* is connected to breast cancer tumorigenesis in relation to the combination of hyperglycemia and hyperinsulinemia, which indeed requires further exploration. On the other hand, Liu et al. described *PABPC4* expression as favorable to patient survival that presented with colorectal cancer [77], whereas the data derived from the Human Protein Atlas indicated that unfavorable outcomes are associated with *PABPC4* expression in both renal cancer and liver cancer [78].

*ALDH1A3* belongs to the family of aldehyde dehydrogenases, which is involved in oxidizing different aldehydes from both exogenous and endogenous sources. The overexpression of these enzymes was noted in hematopoietic and neural stem cells as well as in cancer stem cells [79]. *ALDH1A3* in particular is known for its role in catalyzing the reaction responsible for retinoic acid synthesis, crucial for embryonic and cancer development [80]. Its role in cancer is debatable, since its downregulation was noted in some cancers such as esophageal cancer [81], while its overexpression was linked to tumor resistance and metastasis in pancreatic cancer, gastric and breast cancer [80,82,83,84]. *ALDH1A3* has been shown to initiate breast cancer cell metastasis in the brain; therefore, targeting *ALDH1A3* therapeutically was suggested to improve breast cancer patient survival [85]. *ALDH1A3* overexpression promotes the PI3K/Akt/mTOR pathway; thereby, it elicits its tumorigenic effect [84]. Targeting the *ALDH1A* family of genes by different inhibitors including CM10 showed a reduction in the stemness of ovarian cancer cells [86]. Selective inhibition of ALDH1A3 caused a decline in glioblastoma and colorectal cancer proliferation and invasion [87]. Our data showed that the main metabolic defects, which occur in diabetes, impact the cell machinery by altering *ALDH1A3* gene expression, among others.

*SLC26A11*, the fifth gene that is shared between the in silico and in vitro analyses, is reported as a chloride transporter in the brain and kidney, while being a sulphate transporter in the endothelial venules [88,89,90]. In a genome-wide scale association-based study, the *SLC26A11* gene was found to be differentially expressed in individuals with impaired plasma glucose when compared with the healthy control; this hints to the link between DM-associated metabolic alterations and the *SLC26A11* gene [91]. A different study performed an in silico analysis on data derived from TCGA to study the association of SLC genes with lung adenocarcinoma. Low *SLC26A11* expression was linked to worse overall survival [92]. However, according to the Human Protein Atlas, *SLC26A11* gene expression is unfavorable in both liver and endometrial cancers [93]. The contribution of *SLC26A11* to cancer development is exhibited when a chimeric protein forms by the fusion of RNF213 and *SLC26A11* in CML [94]. Nonetheless, it is apparent that *SLC26A11* has an established link with cancer as well as altered glucose levels. This certainly encourages further research to delineate the role of *SLC26A11* in the interplay between breast cancer and diabetes.

Hyperglycemia has been linked to promoter acetylation, which explains the hyperglycemia-mediated gene upregulation noted in this study [95]. Additionally, hyperinsulinemia caused aberrant histone acetylation in triple negative breast cancer [96]. Conceivably, the combination of hyperglycemia and hyperinsulinemia in the current work has positively affected the promoter activity of different genes, which indeed requires further investigation.

In parallel to the above genes’ expression deregulation, other genes also exhibited changes in their pattern of expression, such as AGER. A report by Yao et al. portrayed that hyperglycemia induces *AGER* expression, which concurs with the DNA microarray data in this study [57]. *AGER* expression is linked with advanced-stage breast cancer [97]. Additional genes with a fold increase of less than two were also overexpressed with HGI treatment, such as *S100P* and *SPINK8*. *S100P* is an intracellular and extracellular calcium-binding protein. Intriguingly, the extracellular *S100P* has the ability to bind RAGE; thereby, it can enhance tumor progression, resistance and migration [98,99]. Therefore, *S100P* is considered a potential therapeutic target and a marker for cancer patients. It is certainly worth determining its levels in diabetic breast cancer patients and correlating its levels with the stage of the disease. Additionally, in this report, *RAGE* showed upregulation, which might exacerbate the oncogenic features of *S100P*. To a lesser extent, *SPINK8* was also slightly increased, and its levels were shown to be elevated in diabetic breast cancer patients as well. Notably, its association with breast cancer as a bad prognostic indicator has been illustrated by the Human Protein Atlas [100].

In the present study, diabetes was taken as the main example of a disease that involves hyperglycemia and hyperinsulinemia as major metabolic alterations; however, individuals with metabolic syndrome, chronic stress and obesity share the same metabolic defects. Therefore, the findings of this study can explain the increased cancer risk that is associated with these metabolic diseases.

Diabetes is a cause of multiorgan dysfunction [101]. It predisposes to retinopathy, nephropathy, neuropathy, endothelial dysfunction, immune incompetence and cancer [102]. Pathological events resulting in most of the stated disorders have been studied comprehensively except for cancer, and little evidence has been revealed. Therefore, providing evidence of how the main metabolic alterations in diabetes, particularly hyperglycemia and hyperinsulinemia, emphasizes the importance of tight glycemic control in diabetic patients with cancer, as uncontrolled blood sugar contributes to worsening patients’ outcomes and overall survival. Moreover, since hyperglycemia and hyperinsulinemia impact the gene expression of tumors, it is worth studying whether they generate a signature that can be considered when diagnosing and treating this group of cancer patients. Finally, hyperglycemia and hyperinsulinemia can be contemplated as a factor in the tumor ecosystem determining temporal tumor heterogeneity.

## 4. Materials and Methods

### 4.1. Study Design

MCF7 cell line was selected in this study since the insulin pathway components have been identified in this cell line. MCF7 cells, despite being obtained from a metastatic site, are well differentiated adenocarcinoma cells, have a moderate replicative ability and do not invade or migrate; consequently, they have been used for many tumor progression studies [103]. Moreover, their response to insulin is well established [104].

**Figure 5:**
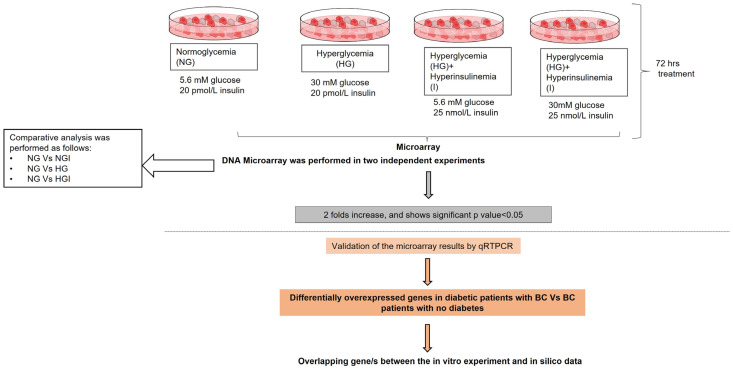
Schematic presentation illustrating the experimental flow.

Accordingly, it was decided to study the effect of high glucose and/or insulin levels on the cancerous behavior of MCF7 cells. MCF7 cell line is a luminal A breast cancer molecular subtype, and it expresses estrogen receptor (ER) and progesterone receptor but not Her2 [105]. It is worth noting that MCF7 cell line expresses insulin receptors as reported by Milazzo G. et al. [106]. First, DNA microarray is employed to identify the differentially expressed genes in hyperglycemia and hyperinsulinemia conditions in comparison to normal glycemia conditions. Subsequently, publicly available breast cancer databases were utilized to delineate the differentially expressed genes in diabetic females with breast cancer versus breast cancer females with normal glycemic index. Accordingly, the genes overlapping between the in vivo observation and in vitro studies were identified by Bioinformatics & Evolutionary Genomics tool [28]. Study design is illustrated in Figure 5.

### 4.2. Deciding Glucose and Insulin Levels

Medium supplemented with 5.6 mM of glucose was used to mimic normoglycemic conditions, while 30 mM was used following previous studies to mimic hyperglycemia [107,108]. Based on the WHO definition of hyperglycemia, 5.6 mM of glucose <11.1 mM is considered a normal glucose level, and levels above that are considered as hyperglycemia [109]. In addition, 30 mM of glucose was proved to have no effect on cell survival after 72 h of exposure in our laboratory [SBM Ahmed and S. Amer, University of Sharjah, Sharjah, UAE (2020), unpublished work].

Insulin concentration that mimics hyperinsulinemia was decided based on a previous study by Mwe-Lin Wei and co-workers, in which they used 25 nM to mimic hyperinsulinemia [25]. According to the reference values of insulin, levels of >174 pmol/L in fasting state as well as >125–1917 pmol/L after 1 h of glucose administration are considered as hyperinsulinemia [110,111,112]. The cells in normoglycemia or hyperglycemia were either left in 20 pmol/L (supplemented in the original medium), which is <174 pmol/L, or 25 nmol/L, which represents hyperinsulinemia. Insulin was purchased from Sigma Aldrich, Gillingham, UK (I 0516).

### 4.3. Cell Culture

The MCF7 cell line was maintained in EMEM (Sigma Aldrich; M2279) supplemented with 10% fetal bovine serum and penicillin (100 U/mL)/streptomycin (100 µg/mL) antibiotic mixture. The cells were kept in a humidified incubator with 5% CO_2_. When maintaining and seeding cells, 1× Trypsin/EDTA was used. Possible aseptic conditions were maintained for all mammalian cell culturing. MCF7 was kindly gifted by Dr. Salem Chouaib of the Institut de Cancérologie Gustave Roussy-Villejuif, France to Sharjah Institute for Medical Research, University of Sharjah, United Arab Emirates.

### 4.4. RNA Extraction

MCF7 cell lines were cultured in different glucose concentrations (5 or 30 mM) for 72 h. The cells were pelleted, and RNA extraction was performed by RNeasy kit (74104) from Qiagen, Hilden, Germany.

RNA quality, labelling and microarray hybridization and microarray data analysis were previously described by Govindaraj et al., 2016 [113]. Below is a brief description of the conducted methods.

### 4.5. RNA Quality Control

A Nanodrop Spectrophotometer was used to evaluate the purity and concentration of the extracted RNA. A bioanalyzer (Agilent; Santa Clara, CA, USA, 2100) was then used to confirm the integrity of the RNA. For this study, good quality RNA was established based on the 260/280 values (Nanodrop, Wilmington, DE, USA), rRNA 28 S/18 S ratios and RNA integrity number (RIN) (Bioanalyzer, Vancouver, BC, Canada).

### 4.6. Labelling and Microarray Hybridization

The Agilent-certified microarray facility of Genotypic Technology, located in Bengaluru, India, performed the microarray hybridization and scanning. The procedure included labelling the samples using Agilent Quick-Amp labelling Kit (p/n5190-0442). Reverse transcription was then conducted on the total RNA at 40 °C using oligo dT primer tagged to a T7 polymerase promoter, yielding double-stranded cDNA. Those synthesized double-stranded cDNAs were used as templates for cRNA generation. This generation needed in vitro transcription along with dye Cy3 CTP(Agilent). Similar to the reverse transcription, the cDNA synthesis and in vitro transcription steps were also carried out at 40 °C. Qiagen RNeasy columns (Qiagen, Cat No: 74106) were used to clean the labelled cRNA. Nanodrop ND-1000 evaluated the quality by assessing the yields and specific activity. Finally, the labelled cRNA samples were fragmented at 60 °C and hybridized onto an Agilent Human Gene Expression Microarray 8 × 60 K. The Gene Expression Hybridization kit (Agilent Technologies, in situ Hybridization kit, Part Number 5190-0404) fragmented the labelled cRNA. Next, hybridization was performed in Agilent’s Surehyb Chambers at 65 °C for 16 h, after which they were washed using Agilent Gene Expression wash buffers (Agilent Technologies, Part Number 5188-5327) and scanned using the Agilent Microarray Scanner (Agilent Technologies, Part Number G2600D).

### 4.7. Microarray Data Analysis

Agilent Feature Extraction software (v11.5.1.1) extracted raw data from the scanned images and fed them into the Agilent GeneSpring GX (v14.5) software for analysis. Normalization was performed using the 75th percentile shift method. In this normalization technique, a multiplicative factor is applied to the entire data, so that the 75th percentile is at the same expression of value for all experiments [40]. Fold change values were obtained by comparing test samples (NGI, HG or HGI) with respect to the control samples (NG) The differentially expressed genes from all conditions (SO-8409_Complete _data_Set 1-4) are available in the Supplementary Materials. Upregulation was defined as having a fold change ≥ 2 (logbase2), while downregulation involved having a fold change ≤−2 (logbase2) for test samples with respect to control sample. Out of the 8 samples, 7 passed the QC, which were then used for the resulting analysis. Normal glycemia control for the second set degraded; therefore, normal glycemia from the first set was utilized for the comparative analysis for both sets. QC reports [QC-DNA Microarray data, Quality Control Report_SO_8409 and SO-8409-repl_Bioanalyzer Report] can be found in the Supplementary Materials.

### 4.8. In Silico Analysis

In this study, we used a publicly deposited transcriptomic dataset of breast cancer patients with or without diabetes (GEO: GSE150586). GSE150586 was obtained by bulk RNA sequencing. In this study, Zhai et al., 2020 performed next generation sequencing to study the differential expression of genes between breast cancer patients and diabetic breast cancer patients [114]. RNA was extracted from 6 breast tumor tissues without diabetes and 7 breast tissues with diabetes and analyzed them using Illumina NovaSeq 6000 (Homo sapiens). Linear models for microarray data (LIMMA) were used to identify the differentially expressed genes between the diabetic group (n = 7) and non-diabetic group (n = 6). The mRNA expression level was presented as fragments per kilobase of exon model per million reads mapped (FPKM).

### 4.9. RT-PCR

Total RNA isolation was performed using RNeasy Mini Kit (Qiagen; 74104). Converting the mRNA to cDNA was performed via RT2 First Strand Kit (Qiagen; 330401). RT-PCR was achieved employing QuantiTect SYBR Green PCR Kit (Qiagen). Housekeeping gene 18 S was used as an internal control. The primers used for RTPCR validation can be found in Supplementary Table S1. The 2^−ΔΔCT^ method was used to calculate the fold change in gene expression.

### 4.10. Statistical Analysis

Benjamini–Hochberg method was used to calculate the adjusted *p*-value for the microarray data. This helped outline the significant genes from the dataset, which is available at Geo with Ref. No. GSE179768. For RTPCR data, one-way ANOVA and Turkey’s multiple comparison test were used to test for change significance between two variables using GraphPad Prism (10.0.0). In silico statistical analysis was performed using R software (v3.0.2) and Prism (v8; GraphPad Software). For all analyses, *p* value < 0.05 was considered significant.

## 5. Limitations

A limitation in the current study is the slight disagreement of RT-PCR with microarray data demonstrated by PAK1, FGFR3 and ALDH1A3, which might be attributed to the differences in the statistical analysis used in these two different techniques [115].

Despite silico-derived data being obtained from a small number of patients, we were able to identify ALDH1A3, MED20, PABPC4, SLC26A11 and SCP2 as deregulated genes in diabetic breast cancer patients, which coincided with our microarray data. Increasing the number of patients in this comparison indeed will yield more conclusive data that reflect how hyperglycemia and hyperinsulinemia influence the gene machinery in tumor cells. These identified genes could also be considered as potential biomarkers as well as therapeutic targets in diabetic breast cancer patients. The publicly available data utilized in this study lacked clinical data, which made it impossible to draw correlation between the cancer stage and the expression levels of the deregulated genes.

Although high insulin levels were linked to cancer development, insulin concentration at the tumor microenvironment has not yet been assessed.

Likewise, to breast cancer molecular subtypes, breast cancer cell lines are classified similarly. The expression of ER/PR among other factors affects the metabolism of breast cancer cell lines [116]; therefore, the effect of glucose might vary between different breast cancer subtypes as well as different breast cancer cell lines, as reported by Farhadi et al., 2022 [116].

The culture conditions are not exactly representative of the in vivo environment. Metabolism is governed by the available nutrients in the medium as well as oxygen levels [117,118], which are not mirrored in an in vitro experiment.

## 6. Conclusions

Cancer is a disease of gene dysregulation. Microenvironment metabolic disruptions such as hyperglycemia and/or hyperinsulinemia can bring about such dysregulation. Patients with these conditions were associated with higher risk of developing breast cancer and exhibit a worse form of the disease. This study revealed how the main metabolic alterations associated with diabetes as well as other metabolic disorders impact the gene expression machinery in tumor cells, which might contribute to tumor advancement and aggressiveness. Future studies are required to delineate the possibility of how this can endorse the personalized treatment concept in cancer patients.

## Figures and Tables

**Table 1:** Differentially upregulated genes induced by the combination of hyperglycemia and hyperinsulinemia treatment.

Gene Name	Average of Fold Change	*p* Value < 0.05	Adj-*p*-Value	Description
*SNRPC*	5.613	<0.001	0.006	Small nuclear ribonucleoprotein polypeptide C
*FGFR3*	3.071	<0.001	0.006	Fibroblast growth factor receptor 3
*FAM219A*	3.469	<0.001	0.005	Family with sequence similarity 219, member A
*SLC26A11*	4.311	<0.001	0.005	Solute carrier family 26 (anion exchanger), member 11
*ATMIN*	2.055	<0.001	0.004	ATM interactor
*ARHGEF10*	3.419	<0.001	0.004	Rho guanine nucleotide exchange factor (GEF) 10
*PAK1*	2.131	<0.001	0.004	p21 protein (Cdc42/Rac)-activated kinase 1
*JPH3*	5.196	<0.001	0.005	Junctophilin 3
*BRD7*	4.668	<0.001	0.005	Bromodomain containing 7
*GNB5*	2.382	<0.001	0.006	Guanine nucleotide binding protein (G protein), beta 5
*LEPR*	5.740	<0.001	0.005	Leptin receptor
*TMEM180*	3.148	<0.001	0.005	Transmembrane protein 180
*SCP2*	3.759	<0.001	0.005	Sterol carrier protein 2
*CDCA5*	4.805	<0.001	0.005	Cell division cycle associated 5
*DYNLT1*	5.364	0.001	0.005	Dynein, light chain, Tctex-type 1
*RBM14*	2.333	0.001	0.005	RNA binding motif protein 14
*GNAZ*	3.279	0.001	0.006	Guanine nucleotide binding protein (G protein), alpha z polypeptide
*STAC3*	2.354	0.001	0.007	SH3 and cysteine rich domain 3
*VKORC1*	4.188	0.001	0.008	Vitamin K epoxide reductase complex, subunit 1
*RNF32*	4.171	0.001	0.008	Ring finger protein 32
*FAM69A*	3.157	0.001	0.008	Family with sequence similarity 69, member A
*MFN2*	4.574	0.001	0.008	Mitofusin 2
*TNFRSF25*	3.608	0.002	0.008	Tumor necrosis factor receptor superfamily, member 25
*DCLRE1C*	2.249	0.002	0.008	DNA cross-link repair 1C
*G3BP1*	5.532	0.002	0.008	GTPase activating protein (SH3 domain) binding protein 1
*TSTD2*	2.449	0.002	0.008	Thiosulfate sulfurtransferase (rhodanese)-like domain containing 2
*PPARD*	2.386	0.002	0.009	Peroxisome proliferator-activated receptor delta
*E2F7*	3.209	0.003	0.010	E2F transcription factor 7
*LRRC1*	3.610	0.003	0.010	Leucine-rich repeat-containing 1
*PABPC4*	4.588	0.003	0.011	Poly(A) binding protein, cytoplasmic 4 (inducible form)
*PAN3*	2.918	0.003	0.011	PAN3 poly(A) specific ribonuclease subunit
*SLITRK6*	2.372	0.003	0.011	SLIT and NTRK-like family, member 6
*LRRC28*	2.118	0.004	0.012	Leucine-rich repeat-containing 28
*NFIA*	2.799	0.004	0.012	Nuclear factor I/A
*LIMD1*	3.078	0.005	0.013	LIM domains containing 1
*AHSA2*	3.386	0.005	0.013	AHA1, activator of heat shock 90kDa protein ATPase homolog 2 (yeast)
*STK3*	3.704	0.005	0.013	Serine/threonine kinase 3
*AGER*	2.129	0.005	0.013	Advanced glycosylation end-product-specific receptor
*TMEM170B*	2.057	0.006	0.013	Transmembrane protein 170B
*PCM1*	3.421	0.006	0.013	Pericentriolar material 1
*KDELC2*	2.870	0.006	0.013	KDEL (Lys-Asp-Glu-Leu) containing 2
*ALDH1A3*	2.978	0.006	0.013	Aldehyde dehydrogenase 1 family, member A3
*MED20*	2.466	0.008	0.015	Mediator complex subunit 20
*SEPN1*	2.196	0.008	0.016	selenoprotein N, 1
*ARAP2*	2.436	0.008	0.016	ArfGAP with RhoGAP domain, ankyrin repeat and PH domain 2
*ZNF550*	2.510	0.009	0.016	Zinc finger protein 550
*HIST2H2BF*	2.396	0.009	0.016	Histone cluster 2, H2bf
*SH3RF3*	2.007	0.013	0.020	SH3 domain-containing ring finger 3
*C3AR1*	2.025	0.014	0.021	Complement component 3a receptor 1
*ZNF808*	2.568	0.014	0.021	Zinc finger protein 808
*DOCK7*	2.394	0.014	0.021	Dedicator of cytokinesis 7
*PLCH1*	2.144	0.014	0.021	Phospholipase C, eta 1
*CLDN12*	2.150	0.017	0.023	Claudin 12
*POU5F1*	2.168	0.018	0.025	POU class 5 homeobox 1
*CEP170*	2.189	0.032	0.036	Centrosomal protein 170kDa
*RAPGEF6*	2.540	0.043	0.045	Rap guanine nucleotide exchange factor (GEF) 6
*CLIP3*	2.340	0.048	0.048	CAP-GLY domain-containing linker protein 3

The results are presented as fold change and normalized to folds of gene expression in cultured cells with normal glucose levels, n = 2. Benjamini–Hochberg method was used to calculate the adjusted *p*-value. MCF7 cells were cultured in 30 mM glucose and 25 nM insulin for 72 h.

## Data Availability

The data produced for this study are either available in this report or in the supplementary data at 0.6084/m9.figshare.2372722. Microarray data is available at GEO accession No. GSE179768.

## Supplementary Materials

The supporting information can be downloaded at: https://www.mdpi.com/article/10.3390/ijms241411816/s1, references [119,120,121,122,123] is cited in Supplementary Materials.
