# Supplementary material for: Assessing the Link between Diabetic Metabolic Dysregulation and Breast Cancer Progression
Source: Int J Mol Sci. 2023 Jul 23;24(14):11816. doi: 10.3390/ijms241411816 (PMC10380477; doi:10.3390/ijms241411816)
Supplement: Supplementary file 1 [file ijms-24-11816-s001.zip › QC_DNA Microarray data.pdf]

Microarray Quality Control Analysis Report

(Genotypic Project No: SO_8409)

For

Dr. Shivang

Genotypic Technology [P] Ltd., #259, "Apurva" 4th Cross, 80 feet road, R.M.V. 2nd Stage, Bangalore-560094,
Ph: +91 80 40538300 & +91 80 23516444 Fax: +91 80 40538222

Index

Content **Page#**

Content	Page#
Microarray Project Plan	3
Total RNA Quality Control	4
Labeling Quality Control	4
Image Quality Control	6

Microarray Project Plan

- **Organism**: Human
- **Slides:** Human 8X60K GXP **AMADID**:072363
- **Starting material**: RNA
- **Labeling kit:** Agilent's Quick-Amp labeling Kit (p/n:5190-0442)
- **Labeling Method:** T7 promoter based-linear amplification to generate labeled complementary RNA (One-Color Microarray-Based Gene Expression Analysis)
- **Total RNA and cRNA Purification Kit:** Qiagen'sRNeasyminikit Cat#74106
- **Hybridization Kit:** Agilent's In situ Hybridzation kit 5190-6420.
- RNA quality was checked using Bioanalyzer

Total RNA Quality Control

Please refer to the folder "**Bioanalyzer_Reports_SO_8409**" containing "**RNA QC report_SO_8409.pdf**" and "**Bioanalyzer Profile SO_8409.pdf**" for samples.

Labeling Quality Control

Labeling QC to check incorporation of Cy3 of labeled RNA

Samples were labeled using the Agilent Quick-Amp labeling Kit. QC was performed using Nanodrop.

Nanodrop Analysis of labeled cRNA

Sample Details	Dye	pmol/µl	ng/µl	260/280	Specific Activity
NG_1	Cy3	0.6	56.8	2.37	10.56
NG_2	Cy3	0.6	65	2.29	9.23
NG+I_1	Cy3	0.9	52.5	2.44	17.14
NG+I_2	Cy3	0.7	62.9	2.26	11.13
HG_1	Cy3	0.6	72.5	1.92	8.28
HG_2	Cy3	0.7	64.4	2.32	10.87
HG+I_1	Cy3	0.7	64.8	2.19	10.80
HG+I_2	Cy3	0.6	49.9	2.2	12.02

Note: Specific Activity greater than 8.0 is Good.
Specific Activity 5.0 to 8.0 is Optimal.

Specific Activity less than 5.0 is not suitable for Hybridization

Comments: The amplified and labeled cRNA is suitable for Hybridization.

Hybridization Design

Slide ID	Sample Hybridised
257236322271_1_1	NG_1
257236322271_1_2	NG_2
257236322271_1_3	NG+I_1
257236322271_1_4	NG+I_2
257236322271_2_1	HG_1
257236322271_2_2	HG_2
257236322271_2_3	HG+I_1
257236322271_2_4	HG+I_2

Image Quality control

Image Quality Control: The images were manually verified and found to be devoid of uneven hybridization, streaks, blobs and other artifacts. Hybridization across the slide was good based on number of feature that were "g is PosAndSignif" which indicates feature is positive and significantly above background.

Comments: The microarray images were overall clean and with very low background noise.

End of Quality Control Report

For queries and clarifications on data analysis report / discuss new projects:

services@genotypic.co.in,contact@genotypic.co.in

Tel: +91- 80- 40538200 AND +91- 80- 23516444 Telefax: +91- 80- 40538222

USA / International Hotline: +1-(215) 253 4495 **Web:** www.genotypic.co.in

Genotypic genomics simplified

RNA Quality Control Report

Genotypic Project No : SO_8409_Repl

Customer name : Dr Samrein Ahmed, University of Sharjah.

Samples Shipped as : Total RNA

No. of samples processed :8

Method of extraction : RNA QC only

RNA Concentration and Purity of samples estimated using Nanodrop Spectrophotometer:

Sl. No.	Sample Name	Date	Absorbance value 260/280	Absorbance value 260/230	RNA Concentration ng/µl	Volume(µl)	Total yield in ng	QC Purity	QC concentration/yield	QC Integrity
1	SO_8409_REPL_1 NG	1/10/2019	2.09	1.62	392.3	50	19615	Optimal	Optimal	Optimal
2	SO_8409_REPL_2 NG	1/10/2019	2.08	1.61	391.8	30	11754	Optimal	Optimal	Degraded
3	SO_8409_REPL_3 NG+I	1/10/2019	2.1	1.45	325.7	45	14656.5	Optimal	Optimal	Optimal
4	SO_8409_REPL_4 NG+I	1/10/2019	2.1	1.93	601.5	40	24060	Optimal	Optimal	Optimal
5	SO_8409_REPL_5 HG	1/10/2019	2.11	1.9	234.7	45	10561.5	Optimal	Optimal	Optimal
6	SO_8409_REPL_6 HG	1/10/2019	2.1	1.84	360.1	45	16204.5	Optimal	Optimal	Optimal
7	SO_8409_REPL_7 HG+I	1/10/2019	2.05	1.9	502.6	45	22617	Optimal	Optimal	Optimal
8	SO_8409_REPL_8 HG+I	1/10/2019	1.59	1.34	517.9	40	20716	Optimal	Optimal	Optimal

An aliquot of the samples was run on an Agilent Bioanalyzer to check for integrity. Please refer to the accompanying file " SO_8409_repl.pdf" for Bioanalyser profiles of the RNA samples.

Comments : 7/8 QC Passed

OPTIMAL: Optimal purity (OD260/280 >1.7 and <2.2; OD260/230>0.5 and <2.5);

Sub-Optimal: Sub-Optimal purity (OD260/280<1.7 and OD 260/230<0.5 and >2.5);

Optimal: optimal concentration (>30 ng/microlitre and <2500 ng/microlitre);

Sub-Optimal: Sub-optimal concentration (<30ng/microlitre);

Note: Sub-optimal purity could lead to failed labeling reaction and sub-optimal concentration can lead to sub-optimal yields of labeled samples.

Assay Class:	Eukaryote Total RNA Nano	Created:	1/14/2019 4:48:19 PM
Comparison	C:\...rt\Comparison\ComparisonFile10_Eukaryote Total RNA Nano.xac	Modified:	1/14/2019 4:48:19 PM

Electropherogram Summary

1NG [SO_8409_Repl]

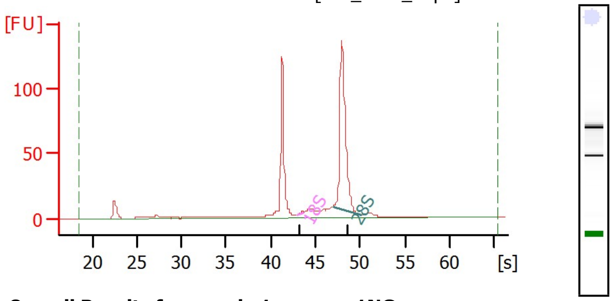

Overall Results for sample 1 : 1NG

RNA Area:	440.9
RNA Concentration:	250 ng/µl
rRNA Ratio [28s / 18s]:	145.7
RNA Integrity Number (RIN):	6.8 (B.02.09)
Result Flagging Color:	
Result Flagging Label:	RIN: 6.80

Fragment table for sample 1 : 1NG

Name	Start Time [s]	End Time [s]	Area	% of total Area
18S	42.91	43.77	1.4	0.3
28S	46.99	50.08	199.9	45.3

2NG [SO_8409_Repl]

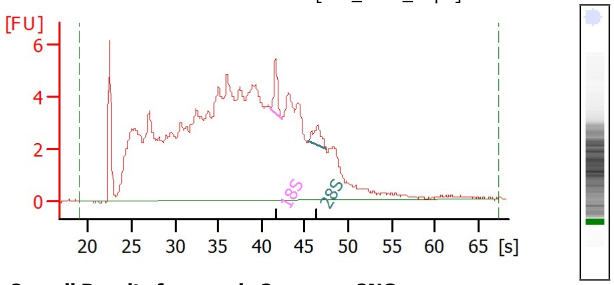

Overall Results for sample 2 : 2NG

RNA Area:	225.9
RNA Concentration:	196 ng/µl
rRNA Ratio [28s / 18s]:	0.8
RNA Integrity Number (RIN):	2.9 (B.02.09)
Result Flagging Color:	
Result Flagging Label:	RIN: 2.90

Fragment table for sample 2 : 2NG

Name	Start Time [s]	End Time [s]	Area	% of total Area
18S	41.05	42.27	2.2	1.0
28S	45.39	47.44	1.7	0.7

3NG+I [SO_8409_Repl]

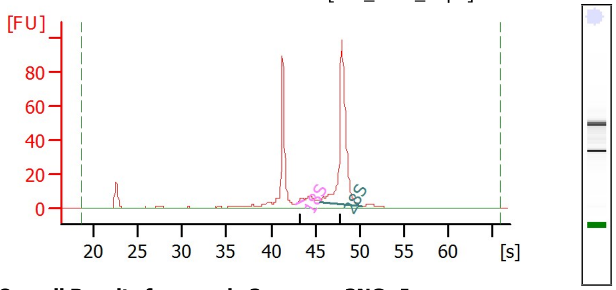

Overall Results for sample 3 : 3NG+I

RNA Area:	356.6
RNA Concentration:	202 ng/µl
rRNA Ratio [28s / 18s]:	86.1
RNA Integrity Number (RIN):	6.7 (B.02.09)
Result Flagging Color:	
Result Flagging Label:	RIN: 6.70

Fragment table for sample 3 : 3NG+I

Name	Start Time [s]	End Time [s]	Area	% of total Area
18S	42.85	43.81	1.9	0.5
28S	45.48	50.16	163.4	45.8

4NG+I [SO_8409_Repl]

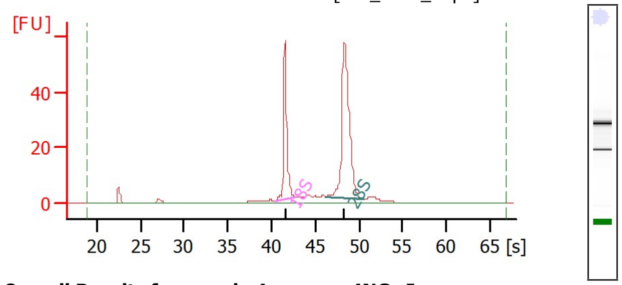

Overall Results for sample 4 : 4NG+I

RNA Area:	238.8
RNA Concentration:	207 ng/µl
rRNA Ratio [28s / 18s]:	2.0
RNA Integrity Number (RIN):	9.9 (B.02.09)
Result Flagging Color:	
Result Flagging Label:	RIN: 9.90

Fragment table for sample 4 : 4NG+I

Name	Start Time [s]	End Time [s]	Area	% of total Area
18S	40.39	42.87	57.4	24.1
28S	46.26	50.48	113.9	47.7

2100 Expert (B.02.09.SI725) © Copyright 2003 - 2017 Agilent Technologies, Inc. Printed: 1/14/2019 4:50:14 PM

Assay Class:	Eukaryote Total RNA Nano	Created:	1/14/2019 4:48:19 PM
Comparison	C:\...rt\Comparison\ComparisonFile10_Eukaryote Total RNA Nano.xac	Modified:	1/14/2019 4:48:19 PM

Electropherogram Summary Continued ...

5HG [SO_8409_Repl]

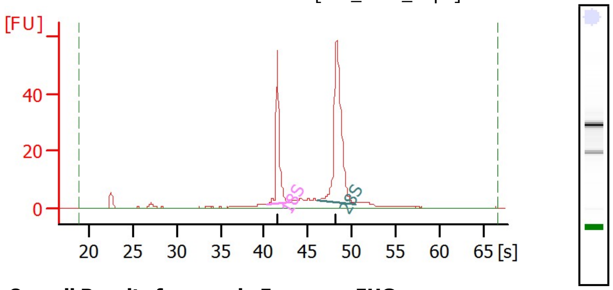

Overall Results for sample 5 : 5HG

RNA Area:	238.6
RNA Concentration:	207 ng/µl
rRNA Ratio [28s / 18s]:	2.0
RNA Integrity Number (RIN):	9.9 (B.02.09)
Result Flagging Color:	
Result Flagging Label:	RIN: 9.90

Fragment table for sample 5 : 5HG

Name	Start Time [s]	End Time [s]	Area	% of total Area
18S	40.40	42.82	54.9	23.0
28S	46.16	50.47	109.3	45.8

6HG [SO_8409_Repl]

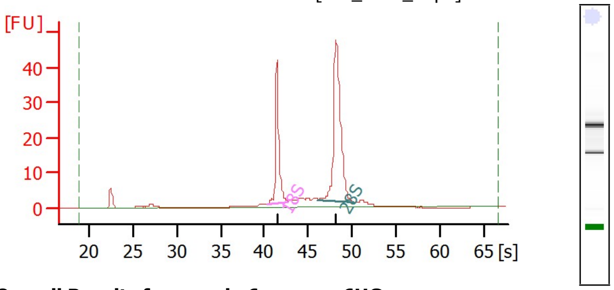

Overall Results for sample 6 : 6HG

RNA Area:	187.2
RNA Concentration:	162 ng/µl
rRNA Ratio [28s / 18s]:	2.1
RNA Integrity Number (RIN):	10 (B.02.09)
Result Flagging Color:	
Result Flagging Label:	RIN:10

Fragment table for sample 6 : 6HG

Name	Start Time [s]	End Time [s]	Area	% of total Area
18S	40.31	42.73	41.8	22.3
28S	46.21	50.23	87.8	46.9

7HG+I [SO_8409_Repl]

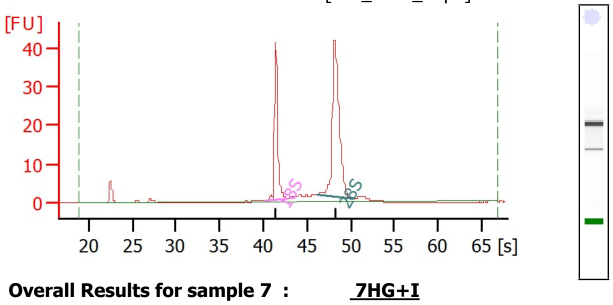

Overall Results for sample 7 : 7HG+I

RNA Area:	157.0
RNA Concentration:	136 ng/µl
rRNA Ratio [28s / 18s]:	2.0
RNA Integrity Number (RIN):	9.9 (B.02.09)
Result Flagging Color:	
Result Flagging Label:	RIN: 9.90

Fragment table for sample 7 : 7HG+I

Name	Start Time [s]	End Time [s]	Area	% of total Area
18S	40.30	42.72	38.4	24.5
28S	46.16	50.29	76.7	48.8

8HG+I [SO_8409_Repl]

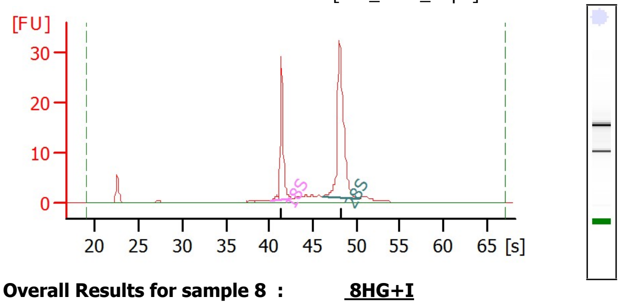

Overall Results for sample 8 : 8HG+I

RNA Area:	111.6
RNA Concentration:	97 ng/µl
rRNA Ratio [28s / 18s]:	2.1
RNA Integrity Number (RIN):	10 (B.02.09)
Result Flagging Color:	
Result Flagging Label:	RIN:10

Fragment table for sample 8 : 8HG+I

Name	Start Time [s]	End Time [s]	Area	% of total Area
18S	40.18	42.56	25.5	22.9
28S	46.07	50.31	54.6	49.0

 © Copyright 2003 - 2017 Agilent Technologies, Inc.

Assay Class:	Eukaryote Total RNA Nano	Created:	1/14/2019 4:48:19 PM
Comparison	C:\...rt\Comparison\ComparisonFile10_Eukaryote Total RNA Nano.xac	Modified:	1/14/2019 4:48:19 PM

Electropherogram Summary Continued ...

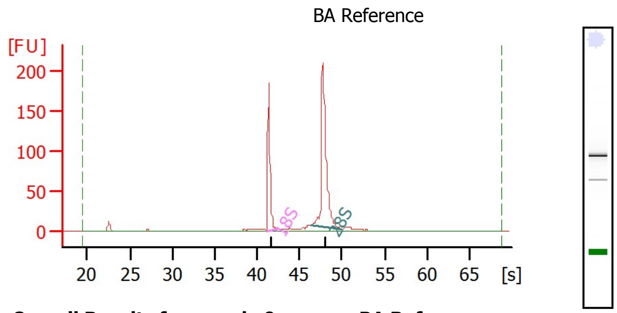

Overall Results for sample 9 : BA Reference

RNA Area:	533.0
RNA Concentration:	489 ng/µl
rRNA Ratio [28s / 18s]:	1.9
RNA Integrity Number (RIN):	10 (B.02.08)
Result Flagging Color:	
Result Flagging Label:	RIN:10

Fragment table for sample 9 : BA Reference

Name	Start Time [s]	End Time [s]	Area	% of total Area
18S	41.06	42.31	140.5	26.4
28S	46.35	49.89	269.8	50.6

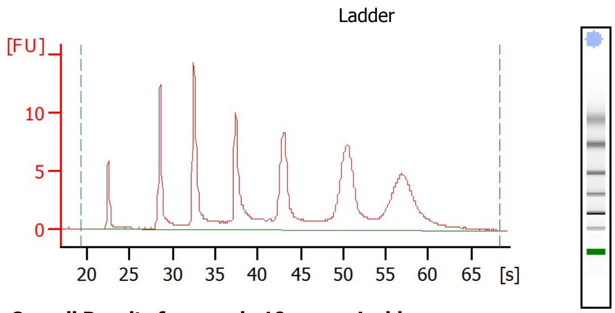

Overall Results for sample 10 : Ladder

RNA Area:	173.2
RNA Concentration:	150 ng/µl
Result Flagging Color:	
Result Flagging Label:	All Other Samples

Fragment table for sample 10 : Ladder

Name	Start Time [s]	End Time [s]	Area	% of total Area
18S	40.18	42.56	25.5	22.9
28S	46.07	50.31	54.6	49.0

 © Copyright 2003 - 2017 Agilent Technologies, Inc.
