# Supplementary material for: Assessing the Link between Diabetic Metabolic Dysregulation and Breast Cancer Progression
Source: Int J Mol Sci. 2023 Jul 23;24(14):11816. doi: 10.3390/ijms241411816 (PMC10380477; doi:10.3390/ijms241411816)
Supplement: Supplementary file 1 [file ijms-24-11816-s001.zip › Quality Control Report_SO_8409.pdf]

# Microarray Quality Control Analysis Report

## (Genotypic Project No: SO_8409)

## For

## Dr. Shivang

Genotypic Technology [P] Ltd., #259, "Apurva" 4th Cross, 80 feet road, R.M.V. 2nd Stage, Bangalore-560094,
Ph: +91 80 40538300 & +91 80 23516444 Fax: +91 80 40538222

# Index

| Content | Page# |
|---|---|
| Microarray Project Plan | 3 |
| Total RNA Quality Control | 4 |
| Labeling Quality Control | 4 |
| Image Quality Control | 6 |

# Microarray Project Plan

- **Organism**: Human
- **Slides:** Human 8X60K GXP **AMADID**:072363
- **Starting material**: RNA
- **Labeling kit:** Agilent's Quick-Amp labeling Kit (p/n:5190-0442)
- **Labeling Method:** T7 promoter based-linear amplification to generate labeled complementary RNA (One-Color Microarray-Based Gene Expression Analysis)
- **Total RNA and cRNA Purification Kit:** Qiagen'sRNeasyminikit Cat#74106
- **Hybridization Kit:** Agilent's In situ Hybridzation kit 5190-6420.
- RNA quality was checked using Bioanalyzer

# Total RNA Quality Control

Please refer to the folder "**Bioanalyzer_Reports_SO_8409**" containing "**RNA QC report_SO_8409.pdf**" and "**Bioanalyzer Profile SO_8409.pdf**" for samples.

# Labeling Quality Control

## Labeling QC to check incorporation of Cy3 of labeled RNA

Samples were labeled using the Agilent Quick-Amp labeling Kit. QC was performed using Nanodrop.

### Nanodrop Analysis of labeled cRNA

| Sample Details | Dye | pmol/µl | ng/µl | 260/280 | Specific Activity |
|---|---|---|---|---|---|
| NG_1 | Cy3 | 0.6 | 56.8 | 2.37 | 10.56 |
| NG_2 | Cy3 | 0.6 | 65 | 2.29 | 9.23 |
| NG+I_1 | Cy3 | 0.9 | 52.5 | 2.44 | 17.14 |
| NG+I_2 | Cy3 | 0.7 | 62.9 | 2.26 | 11.13 |
| HG_1 | Cy3 | 0.6 | 72.5 | 1.92 | 8.28 |
| HG_2 | Cy3 | 0.7 | 64.4 | 2.32 | 10.87 |
| HG+I_1 | Cy3 | 0.7 | 64.8 | 2.19 | 10.80 |
| HG+I_2 | Cy3 | 0.6 | 49.9 | 2.2 | 12.02 |

**Note: Specific Activity greater than 8.0 is Good.**
**Specific Activity 5.0 to 8.0 is Optimal.**

**Specific Activity less than 5.0 is not suitable for Hybridization**

**Comments:** **The amplified and labeled cRNA is suitable for Hybridization.**

# Hybridization Design

| Slide ID | Sample Hybridised |
|---|---|
| 257236322271_1_1 | NG_1 |
| 257236322271_1_2 | NG_2 |
| 257236322271_1_3 | NG+I_1 |
| 257236322271_1_4 | NG+I_2 |
| 257236322271_2_1 | HG_1 |
| 257236322271_2_2 | HG_2 |
| 257236322271_2_3 | HG+I_1 |
| 257236322271_2_4 | HG+I_2 |

# Image Quality control

**Image Quality Control:** The images were manually verified and found to be devoid of uneven hybridization, streaks, blobs and other artifacts. Hybridization across the slide was good based on number of feature that were "g is PosAndSignif" which indicates feature is positive and significantly above background.

Comments: The microarray images were overall clean and with very low background noise.

---

**End of Quality Control Report**

**For queries and clarifications on data analysis report / discuss new projects:**

services@genotypic.co.in,contact@genotypic.co.in

**Tel: +91- 80- 40538200 AND +91- 80- 23516444 Telefax: +91- 80- 40538222**

**USA / International Hotline: +1-(215) 253 4495** **Web:** www.genotypic.co.in
