# Supplementary material for: Assessing the Link between Diabetic Metabolic Dysregulation and Breast Cancer Progression
Source: Int J Mol Sci. 2023 Jul 23;24(14):11816. doi: 10.3390/ijms241411816 (PMC10380477; doi:10.3390/ijms241411816)
Supplement: Supplementary file 1 [file ijms-24-11816-s001.zip › SO_8409_repl_Bioanalyzer Report.pdf]

Assay Class:	Eukaryote Total RNA Nano	Created:	1/14/2019 4:48:19 PM
Comparison	C:\...rt\Comparison\ComparisonFile10_Eukaryote Total RNA Nano.xac	Modified:	1/14/2019 4:48:19 PM

Electropherogram Summary

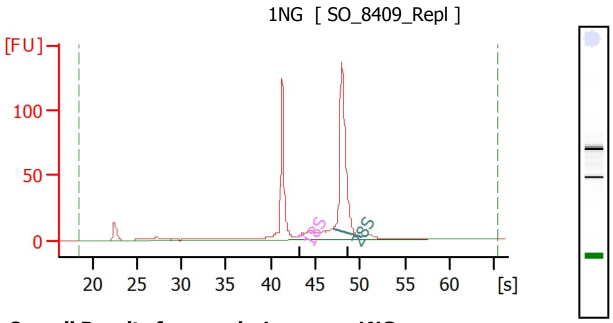

Overall Results for sample 1 : 1NG

RNA Area:	440.9
RNA Concentration:	250 ng/µl
rRNA Ratio [28s / 18s]:	145.7
RNA Integrity Number (RIN):	6.8 (B.02.09)
Result Flagging Color:	
Result Flagging Label:	RIN: 6.80

Fragment table for sample 1 : 1NG

Name	Start Time [s]	End Time [s]	Area	% of total Area
18S	42.91	43.77	1.4	0.3
28S	46.99	50.08	199.9	45.3

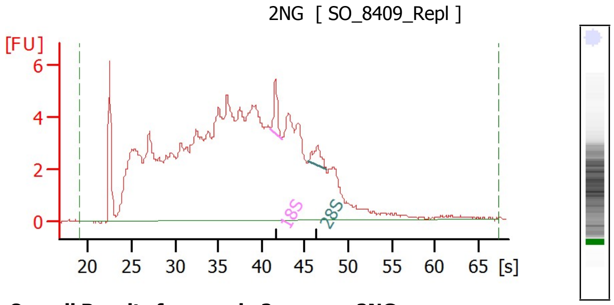

Overall Results for sample 2 : 2NG

RNA Area:	225.9
RNA Concentration:	196 ng/µl
rRNA Ratio [28s / 18s]:	0.8
RNA Integrity Number (RIN):	2.9 (B.02.09)
Result Flagging Color:	
Result Flagging Label:	RIN: 2.90

Fragment table for sample 2 : 2NG

Name	Start Time [s]	End Time [s]	Area	% of total Area
18S	41.05	42.27	2.2	1.0
28S	45.39	47.44	1.7	0.7

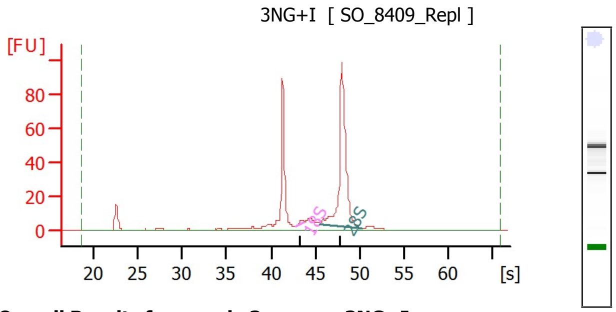

Overall Results for sample 3 : 3NG+I

RNA Area:	356.6
RNA Concentration:	202 ng/µl
rRNA Ratio [28s / 18s]:	86.1
RNA Integrity Number (RIN):	6.7 (B.02.09)
Result Flagging Color:	
Result Flagging Label:	RIN: 6.70

Fragment table for sample 3 : 3NG+I

Name	Start Time [s]	End Time [s]	Area	% of total Area
18S	42.85	43.81	1.9	0.5
28S	45.48	50.16	163.4	45.8

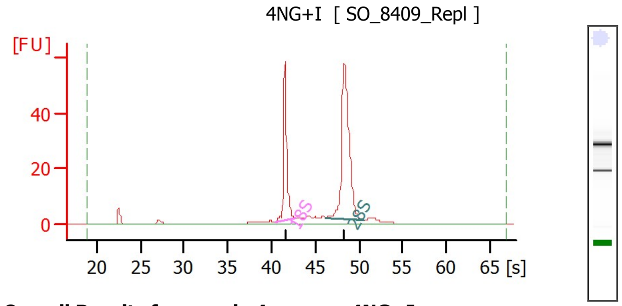

Overall Results for sample 4 : 4NG+I

RNA Area:	238.8
RNA Concentration:	207 ng/µl
rRNA Ratio [28s / 18s]:	2.0
RNA Integrity Number (RIN):	9.9 (B.02.09)
Result Flagging Color:	
Result Flagging Label:	RIN: 9.90

Fragment table for sample 4 : 4NG+I

Name	Start Time [s]	End Time [s]	Area	% of total Area
18S	40.39	42.87	57.4	24.1
28S	46.26	50.48	113.9	47.7

 © Copyright 2003 - 2017 Agilent Technologies, Inc.

Assay Class: Eukaryote Total RNA Nano
Comparison C:\...rt\Comparison\ComparisonFile10_Eukaryote Total RNA Nano.xac
Created: 1/14/2019 4:48:19 PM
Modified: 1/14/2019 4:48:19 PM

Electropherogram Summary Continued ...

5HG [SO_8409_Repl]

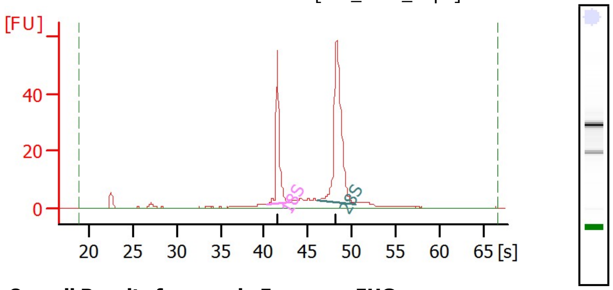

Overall Results for sample 5 : 5HG

RNA Area:	238.6
RNA Concentration:	207 ng/µl
rRNA Ratio [28s / 18s]:	2.0
RNA Integrity Number (RIN):	9.9 (B.02.09)
Result Flagging Color:	
Result Flagging Label:	RIN: 9.90

Fragment table for sample 5 : 5HG

Name	Start Time [s]	End Time [s]	Area	% of total Area
18S	40.40	42.82	54.9	23.0
28S	46.16	50.47	109.3	45.8

6HG [SO_8409_Repl]

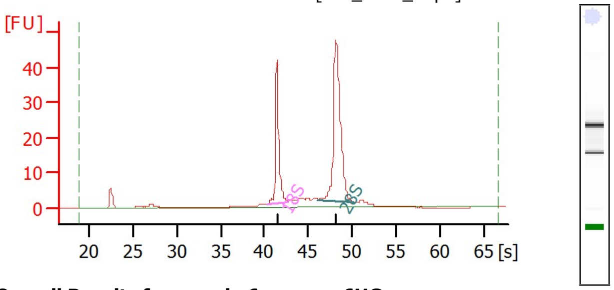

Overall Results for sample 6 : 6HG

RNA Area:	187.2
RNA Concentration:	162 ng/µl
rRNA Ratio [28s / 18s]:	2.1
RNA Integrity Number (RIN):	10 (B.02.09)
Result Flagging Color:	
Result Flagging Label:	RIN:10

Fragment table for sample 6 : 6HG

Name	Start Time [s]	End Time [s]	Area	% of total Area
18S	40.31	42.73	41.8	22.3
28S	46.21	50.23	87.8	46.9

7HG+I [SO_8409_Repl]

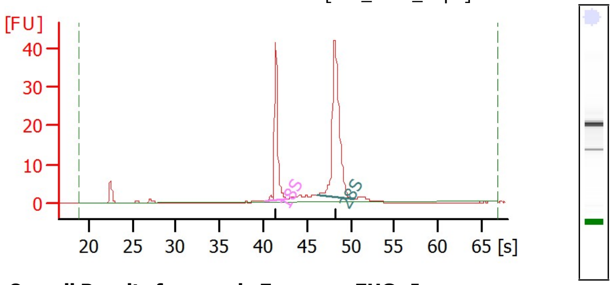

Overall Results for sample 7 : 7HG+I

RNA Area:	157.0
RNA Concentration:	136 ng/µl
rRNA Ratio [28s / 18s]:	2.0
RNA Integrity Number (RIN):	9.9 (B.02.09)
Result Flagging Color:	
Result Flagging Label:	RIN: 9.90

Fragment table for sample 7 : 7HG+I

Name	Start Time [s]	End Time [s]	Area	% of total Area
18S	40.30	42.72	38.4	24.5
28S	46.16	50.29	76.7	48.8

8HG+I [SO_8409_Repl]

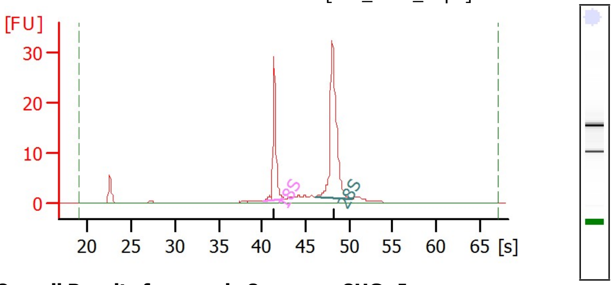

Overall Results for sample 8 : 8HG+I

RNA Area:	111.6
RNA Concentration:	97 ng/µl
rRNA Ratio [28s / 18s]:	2.1
RNA Integrity Number (RIN):	10 (B.02.09)
Result Flagging Color:	
Result Flagging Label:	RIN:10

Fragment table for sample 8 : 8HG+I

Name	Start Time [s]	End Time [s]	Area	% of total Area
18S	40.18	42.56	25.5	22.9
28S	46.07	50.31	54.6	49.0

© Copyright 2003 - 2017 Agilent Technologies, Inc.

Assay Class: Eukaryote Total RNA Nano
Comparison C:\...rt\Comparison\ComparisonFile10_Eukaryote Total RNA Nano.xac

Created: 1/14/2019 4:48:19 PM
Modified: 1/14/2019 4:48:19 PM

Electropherogram Summary Continued ...

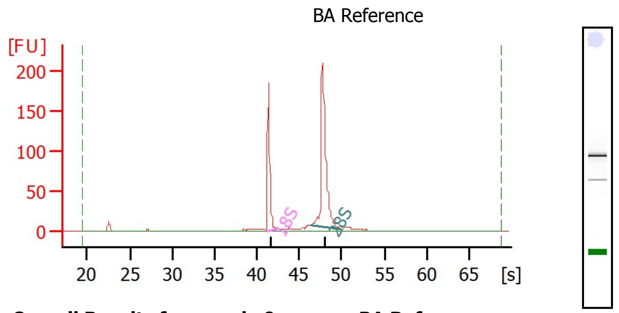

Overall Results for sample 9 : **BA Reference**

RNA Area:	533.0
RNA Concentration:	489 ng/µl
rRNA Ratio [28s / 18s]:	1.9
RNA Integrity Number (RIN):	10 (B.02.08)
Result Flagging Color:	
Result Flagging Label:	RIN:10

Fragment table for sample 9 : **BA Reference**

Name	Start Time [s]	End Time [s]	Area	% of total Area
18S	41.06	42.31	140.5	26.4
28S	46.35	49.89	269.8	50.6

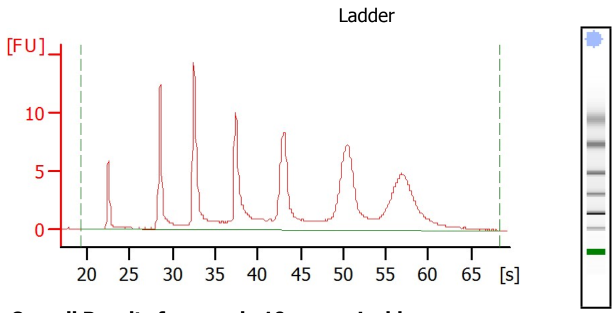

Overall Results for sample 10 : **Ladder**

RNA Area:	173.2
RNA Concentration:	150 ng/µl
Result Flagging Color:	
Result Flagging Label:	All Other Samples

Fragment table for sample 10 : **Ladder**

Name	Start Time [s]	End Time [s]	Area	% of total Area
18S	40.18	42.56	25.5	22.9
28S	46.07	50.31	54.6	49.0

 © Copyright 2003 - 2017 Agilent Technologies, Inc.
