# Supplementary material for: Assessing the Link between Diabetic Metabolic Dysregulation and Breast Cancer Progression
Source: Int J Mol Sci. 2023 Jul 23;24(14):11816. doi: 10.3390/ijms241411816 (PMC10380477; doi:10.3390/ijms241411816)
Supplement: Supplementary file 1 [file ijms-24-11816-s001.zip › Table 1S, 2S, 3S and 4S.pdf]

Table 1 Primers sequence used for RTPCR.

	Gene name	Forward	Reverse	Citation	Annealing temperature
1	PAK1	GGTGGTGGCTGCACAGTAG	TCTGAGGCAGGAGGTGGTAA	(1)	58°C
2	ALDH1A3	TGGATCAACTGCTACAACGC	CACTTCTGTGTATTCGGCCA	(2)	60°C
3	FGFR3	CCTCGGGAGATGACGAAGAC	CGGGCCGTGTCCAGTAAGG	(3)	60°C
4	CDCA5	ATC CAC CTC GCA GGA GCC CTA	CTC TCC TTC CTT GGA GCT GGA	(4)	60°C
5	TGFA	CCG AGG CAG TCA GAT CAT CTT	AGC TGC CCC TCA GCT TGA	(5)	60°C

Table 2S Differentially upregulated genes by hyperglycemia treatment

Gene Symbol	Average of the fold	P_Value	Gene
PAN3	2.794	0.000	PAN3 poly(A) specific ribonuclease subunit
CDCA5	4.833	0.000	cell division cycle associated 5
ZNF550	2.424	0.001	zinc finger protein 550
OLA1	2.789	0.001	Obg-like ATPase 1
TSTD2	2.356	0.001	thiosulfate sulfurtransferase (rhodanese)-like domain containing 2
CALML4	2.308	0.001	calmodulin-like 4
STAC3	2.435	0.001	SH3 and cysteine rich domain 3
FAM219A	3.515	0.001	family with sequence similarity 219, member A
MUC3A	2.360	0.002	mucin 3A, cell surface associated
TINAGL1	2.085	0.002	tubulointerstitial nephritis antigen-like 1
DCLRE1C	2.059	0.003	DNA cross-link repair 1C
CBX2	4.939	0.003	chromobox homolog 2
TMEM71	2.657	0.006	transmembrane protein 71
SLITRK6	2.578	0.008	SLIT and NTRK-like family, member 6
TGFA	2.111	0.011	transforming growth factor, alpha
C3AR1	2.029	0.013	complement component 3a receptor 1

The results are presented as fold change and normalized to folds of gene expression in normal glucose levels cultured cells. N=2

MCF7 cells were cultured in 30 mM glucose for 72 hrs.

Table 3S Differentially upregulated genes by hyperinsulinemia treatment

Gene Name	Folds' average	P_Value	Description
TGFA	2.195	0.000	transforming growth factor, alpha
ECH1	4.088	0.000	enoyl CoA hydratase 1, peroxisomal
CALML4	2.595	0.000	calmodulin-like 4

CDCA5	4.814	0.000	cell division cycle associated 5
TSTD2	2.477	0.001	thiosulfate sulfurtransferase (rhodanese)-like domain containing 2
DCLRE1C	2.251	0.001	DNA cross-link repair 1C
MUC3A	2.405	0.001	mucin 3A, cell surface associated
C19orf68	3.667	0.002	chromosome 19 open reading frame 68
PCM1	3.302	0.002	pericentriolar material 1
PAN3	2.709	0.003	PAN3 poly(A) specific ribonuclease subunit
FAM69A	2.874	0.003	family with sequence similarity 69, member A
LIMD1	2.877	0.003	LIM domains containing 1
GRIK2	2.775	0.004	glutamate receptor, ionotropic, kainate 2
HIF3A	2.003	0.005	hypoxia inducible factor 3, alpha subunit
STAC3	2.604	0.006	SH3 and cysteine rich domain 3
DDIT3	2.786	0.009	DNA-damage-inducible transcript 3
AKR1B1	2.048	0.017	aldo-keto reductase family 1, member B1 (aldose reductase)
ZC3H12D	2.053	0.040	zinc finger CCCH-type containing 12D

The results are presented as fold change and normalized to folds of gene expression in normal glucose levels cultured cells. N=2

MCF7 cells were cultured in 25 nM insulin for 72 hrs.

Table 4S Differentially upregulated genes shared between different treatments.

HGI/NGI	HG/HGI	NGI/HG	HG/HGI/NGI
PCM1	ZNF55	TGFA	PAN3
FAM69A	FAM219A	CALML4	CDCA5
LIMD1	SLITRK6	MUC3A	TSTD2
	C3AR1		STAC3
			DCRE1C

References:

1. Fu H, Zhang W, Yuan Q, Niu M, Zhou F, Qiu Q, et al. PAK1 Promotes the Proliferation and Inhibits Apoptosis of Human Spermatogonial Stem Cells via PDK1/KDR/ZNF367 and ERK1/2 and AKT Pathways. Mol Ther Nucleic Acids. 2018;12:769-86.
2. Sullivan KE, Rojas K, Cerione RA, Nakano I, Wilson KF. The stem cell/cancer stem cell marker ALDH1A3 regulates the expression of the survival factor tissue transglutaminase, in mesenchymal glioma stem cells. Oncotarget. 2017;8(14):22325-43.
3. Tomlinson DC, L'Hôte CG, Kennedy W, Pitt E, Knowles MA. Alternative splicing of fibroblast growth factor receptor 3 produces a secreted isoform that inhibits fibroblast growth factor-induced proliferation and is repressed in urothelial carcinoma cell lines. Cancer Res. 2005;65(22):10441-9.
4. Tokuzen N, Nakashiro K, Tanaka H, Iwamoto K, Hamakawa H. Therapeutic potential of targeting cell division cycle associated 5 for oral squamous cell carcinoma. Oncotarget. 2016;7(3):2343-53.
5. Carneiro JR, Fuzii HT, Kayser C, Alberto FL, Soares FA, Sato EI, et al. IL-2, IL-5, TNF-α and IFN-γ mRNA expression in epidermal keratinocytes of systemic lupus erythematosus skin lesions. Clinics (Sao Paulo). 2011;66(1):77-82.
